# Risk of chronic Q fever in patients with cardiac valvulopathy, seven years after a large epidemic in the Netherlands

**DOI:** 10.1371/journal.pone.0221247

**Published:** 2019-08-22

**Authors:** Marit M. A. de Lange, Arko Scheepmaker, Wim van der Hoek, Monique Leclercq, Peter M. Schneeberger

**Affiliations:** 1 Centre for Infectious Disease Control Netherlands, National Institute for Public Health and the Environment, Bilthoven, the Netherlands; 2 Department of Cardiology, Bernhoven Hospital, Uden, the Netherlands; 3 Department of Internal Medicine, Bernhoven Hospital, Uden, the Netherlands; 4 Department of Medical Microbiology, Jeroen Bosch Hospital, ‘s-Hertogenbosch, the Netherlands; Oregon Health & Science University, UNITED STATES

## Abstract

**Background:**

From 2007 through 2010, a large epidemic of acute Q fever occurred in the Netherlands. Patients with cardiac valvulopathy are at high risk to develop chronic Q fever after an acute infection. This patient group was not routinely screened, so it is unknown whether all their chronic infections were diagnosed. This study aims to investigate how many chronic Q fever patients can be identified by routinely screening patients with valvulopathy and to establish whether the policy of not screening should be changed.

**Methods:**

In a cross-sectional study (2016–2017) in a hospital at the epicentre of the Q fever epidemic, a blood sample was taken from patients 18 years and older who presented with cardiac valvulopathy. The sample was tested for IgG antibodies against phase I and II of *Coxiella burnetii* using an immunofluorescence assay. An IgG phase II titre of ≥1:64 was considered serological evidence of a previous Q fever infection. An IgG phase I titre of ≥1:512 was considered suspicious for a chronic infection, and these patients were referred for medical examination.

**Results:**

Of the 904 included patients, 133 (15%) had evidence of a previous *C*. *burnetii* infection, of whom 6 (5%) had a chronic infection on medical examination.

**Conclusions:**

In a group of high-risk patients with a heart valve defect, we diagnosed new chronic Q fever infections seven years after the epidemic, emphasizing the need for screening of this group to prevent complications in those not yet diagnosed in epidemic areas.

## Introduction

In the Netherlands, a large epidemic of Q fever occurred from 2007 through 2010, with more than 4,000 reported acute Q fever patients [1], whose most common clinical presentation was pneumonia. [2] These 4,000 reported cases are estimated to reflect more than 50,000 acute infections with *Coxiella burnetii*, including asymptomatic patients and symptomatic patients not seeking medical care or not being diagnosed with *C*. *burnetii* infection. [3]

Chronic Q fever can develop in 5% of all symptomatic acute Q fever patients. [4] A serious disease with high morbidity and mortality, it most often presents in patients with risk factors such as cardiac valve and vascular disease or immunodeficiency. [5–7] Long-term treatment with antibiotics of at least 18 months, consisting of the combination of doxycycline and hydroxychloroquine, and cardiovascular surgical procedures can improve the prognosis. [7–9] Predominant clinical presentations of chronic Q fever are endocarditis and endovascular infection. [5–7] In the aftermath of the Dutch epidemic, more vascular chronic infections were diagnosed, compared to endocarditis. [10] However, in the south of France, where much research on chronic Q fever has been performed, the opposite is seen: more endocarditis is diagnosed than vascular chronic infection. In the Netherlands to date, only patients with a history of valvular replacement were screened for chronic Q fever, in only one hospital [11]. The entire group of patients with valvulopathy, irrespective of surgical treatment, has not been screened and therefore, chronic *C*. *burnetii* infections may have been missed or diagnosed late.

The objective of this study is to investigate how many chronic Q fever patients can still be identified, several years after the epidemic, by routinely screening of patients with valvulopathy in the high incidence area. This finding will be important to inform policy on screening during future Q fever outbreaks.

## Methods

### Patient enrolment

**Fig 1:**
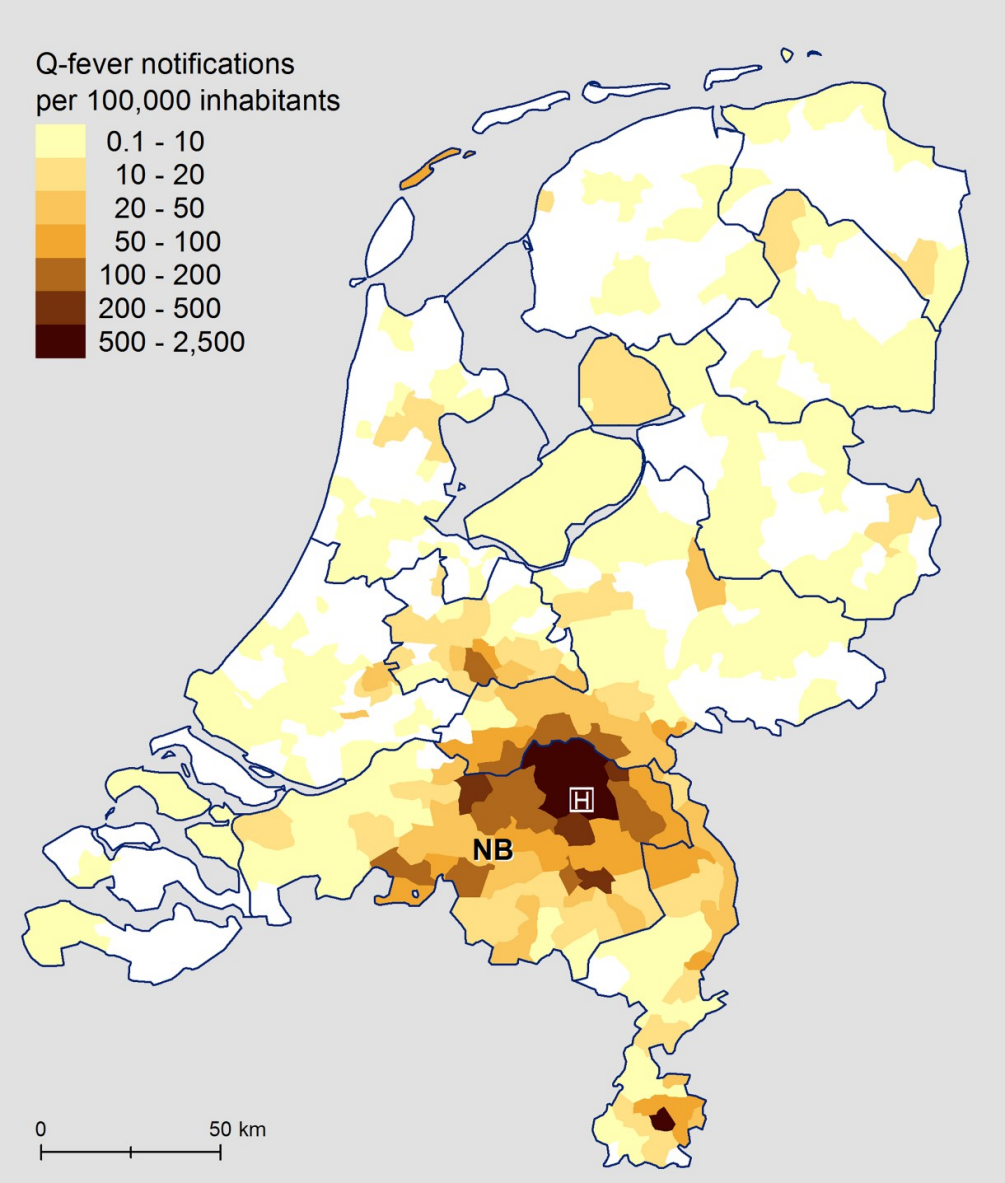
Q fever notifications per 100,000 inhabitants with location of Bernhoven hospital footnote. NB = province Noord-Brabant. Source of the data: Notification system (OSIRIS), and Statistics Netherlands (CBS).

The study was performed in the Bernhoven hospital, which is located in the small town of Uden, in the centre of the North Brabant province, where Q fever was epidemic (Fig 1). This hospital has a catchment area of around 300.000 people. Over a one-year period (15 February 2016 through 17 February 2017), patients aged 18 years and older were eligible for inclusion if newly diagnosed with or already known to have a valvulopathy at the cardiology outpatient clinic, or who were admitted to the cardiology ward. We invited patients with a mild, moderate, or severe insufficiency or stenosis of aortic or mitral valves that were natural or artificial. The eligible patients received the following study documents: information letter, a laboratory form for the blood collection, and an informed consent letter. We asked the participants for permission to examine their electronic patient records for possible risk factors for chronic infection (age, gender, postal code area, cardiac and non-cardiac medical conditions). All participants provided their written consent to participate in this study. We excluded patients already known to have chronic Q fever infection.

The Medical Ethics Review Committee of Brabant examined the study protocol and concluded that the Medical Research Involving Human Subjects Act (WMO) was not applicable and that the study complies with the Data Protection Act (WBP), the Medical Treatment Contracts Act (WGBO), and the Code of Conduct for Health Research, and the Code of Conduct for Responsible Use. See S1 and S2 Files for the translated documents of the Ethics Review Committee. Additionally, the management board of the Bernhoven hospital approved the local feasibility.

### Echocardiography

The eleven cardiologists of the Bernhoven hospital interpreted all echocardiographs according to the European Society of Cardiology (ESC) guidelines. [12] The aortic and mitral valves were examined for regurgitation and stenosis. Echocardiographic results were retrieved from electronic patient records by one of the authors (MMAdL) and support staff at the cardiology department. If the description was not clear, the researcher consulted one of the cardiologists (AS). Per diagnosis, patients were subdivided into groups with no, mild, moderate, or severe valvulopathy.

### Diagnosis

We performed screening on serum samples obtained by venepuncture. First, sera were screened for IgG antibodies against phase I and II of *C*. *burnetii* using an immunofluorescence assay (IFA; Focus Diagnostics, INC., Cypress, CA, USA) with a detection cut-off titre of ≥1:64. We considered patients with a phase II IgG titre of ≥1:64 to have serological evidence of a previous *C*. *burnetii* infection. An IgG phase I titre of ≥1:512 was suspicious for a chronic Q fever infection. If phase I IgG antibodies were present at or above this cut-off, we determined the exact antibody titre. We performed real-time PCR for *C*. *burnetii* DNA if the phase I IgG titre was ≥1:512. [13] This is below the chronic Q fever definition cut-off titre of ≥1:1024 (see next paragraph) and was chosen to increase the probability of capturing all cases. Participants with antibodies present at or above 1:512 were advised to be referred to the Internal Medicine Department in the Bernhoven Hospital for further examination. Eight months after the last patient was included, we checked the outcome of those who were further evaluated because of an IgG phase I titre ≥1:512. As suggested by the Dutch Q fever Consensus group, we categorised the chronic Q fever infections into probable or proven chronic Q fever. All participants with an IgG phase I titre of ≥1:1024 against *C*. *burnetii* were classified as probable cases of chronic Q fever, as they all possess a risk factor for a chronic infection, namely a valvulopathy. If additionally the patient had a PCR positive for *C*. *burnetii* in blood or tissue, had a definite endocarditis according to the modified Duke criteria, or had a proven large vessel or prosthetic infection, then they were classified as having a proven chronic Q fever infection (S1 Table). [14]

### Data analysis

We calculated frequencies and percentages for the baseline characteristics. Next, we performed univariable logistic regression to estimate possible risk factors for chronic infection, compared to patients who had serological evidence of a previous *C*. *burnetii* infection but had no chronic Q fever infection. We considered age, gender, various health complaints/diseases, and heart valve diseases as possible risk factors. Additionally, we performed univariable logistic regression to estimate possible risk factors for chronic infection, compared to those who had no chronic infection (patients who had serological evidence of a previous *C*. *burnetii* infection but had no chronic Q fever infection and seronegative patients taken together). The small number of identified chronic infections precluded multivariable analysis. We performed all analyses using SAS software version 9.4 (SAS institute, Cary, North Carolina, USA).

## Results

**Fig 2:**
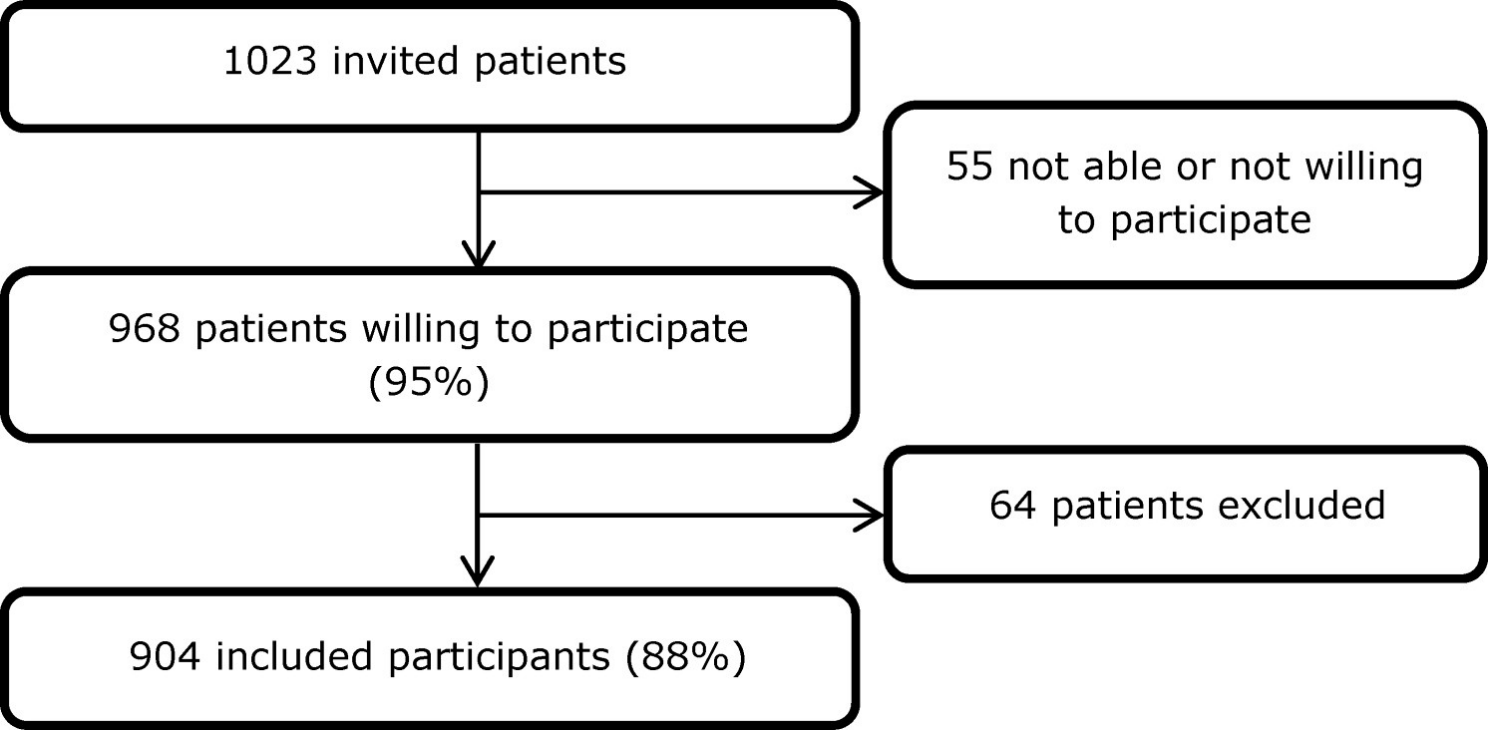
Flow diagram participant inclusion.

We invited 1023 people for the study, of whom 968 were willing to participate (Fig 2). Of those 968 persons, 64 were excluded because inclusion or blood sampling was not performed according to the study protocol; because valvulopathy was not found on reviewing the echocardiographic results; or because the echocardiographic images could not be retrieved. Therefore, 904 patients were included in the analysis. None was already known to have a chronic Q fever infection. Of the 904 participants, 133 (15%) had serological evidence of a previous *C*. *burnetii* infection, as they had an IgG phase II titre of 1:64 or higher. Of these 133 participants, 11 had a phase I titre of 1:512 or higher. Of these, further clinical examination at the Internal Medicine Department showed that five had no chronic infection, as they had an IgG phase I titre of 1:512 and no other signs of chronic infection. The other six (5%) were diagnosed with a chronic Q fever infection. See S1 Dataset for the anonymised data of the 904 participants.

Table 1 shows the baseline characteristics of the complete cohort. Baseline characteristics are presented in S2 Table separately for patients with no previous *C*. *burnetii* infection, patients with serological evidence of a previous *C*. *burnetii* infection but no chronic Q fever infection, and patients with a chronic infection. Patients with chronic Q fever had a higher prevalence of COPD, vascular prosthesis of the large body vessels, and vascular abnormalities of the large body vessels than persons with or without serological evidence of a previous *C*. *burnetii* infection.

**Table 1:** Characteristics of the study participants^a^.

Characteristic		
Gender	n/N (% male)	487/904 (54)
Age	Median (min–max) (years)	73 (26–95)
Diabetes	n/N (%)	157/886 (18)
COPD	n/N (%)	80/886 (9)
Asthma	n/N (%)	40/886 (5)
Impaired kidney function or chronic kidney disease ^b^	n/N (%)	238/886 (27)
Stroke	n/N (%)	60/886(7)
Hematologic cancer ^c^	n/N (%)	4/886 (<1)
Cancer, other than hematologic cancer ^c^	n/N (%)	90/886 (10)
Autoimmune disease	n/N (%)	107/886 (12)
HIV	n/N (%)	0/886 (0)
Vascular prosthesis of the large body vessels ^d^	n/N (%)	30/886 (3)
Vascular abnormality of the large body vessels ^e^	n/N (%)	86/886 (10)

Abbreviations: n = Number, N = total number

^a^ 18 Participants gave no permission to collect data from the electronic patient record.

^b^ Estimated kidney function (modification of diet in renal disease) in majority of tests in recent years smaller than 60.

^c^ Cancer present in last five years before inclusion.

^d^ Vascular prosthesis of the aorta, femoral artery, or common iliac artery.

^e^ Aneurysm or vascular dilatation of the aortic arch (>29mm) or ascending aorta (>40 mm) described in echocardiographic report or dilatation of abdominal aorta, femoral artery, or common iliac artery mentioned in the electronic patient record.

Table 2 provides details of the six chronic Q fever patients. Three had a proven chronic infection, of which two had an infection of the aortic tube, and one an endocarditis. Two patients had a probable chronic infection, as they had an IgG phase I titre ≥1:1024 with valvulopathy as risk factor but had no evidence of actual chronic infection. One patient with high IgG phase I titre did not want to be referred to the Internal Medicine Department, as he experienced no disease symptoms, so the classification of his infection remained unknown. Only one of the six patients was aware of a previous acute *C*. *burnetii* infection, having participated in an earlier Q fever serosurvey.

**Table 2:** Characteristics of the six participants with a chronic infection.

Patient	IgG phase I	IgG phase II	Gender	Age	Underlying disease	Clinical presentation
1	1:8192	1:8192	Male	76	Moderate mitral regurgitation	Probable chronic Q fever, no focus discovered
2	1:4096	1:4096	Male	81	Mild mitral regurgitation, aortic tube prosthesis	Proven chronic Q fever, aortic tube infection localised
3	1:4096	1:4096	Male	83	Moderate mitral regurgitation, aortic prosthesis valve, impaired kidney function	Proven chronic Q fever, abdominal aortic infection localised
4	1:2048	1:2048	Male	61	Mild aortic regurgitation and moderate aortic stenosis of bicuspid valve, aneurysm of aorta ascendens	Proven chronic Q fever, endocarditis
5	1:2048	1:4096	Male	83	Mild mitral regurgitation	Unknown chronic Q fever status
6	1:512 ^a^	1:1024	Female	68	Moderate mitral regurgitation, COPD GOLD 1, aneurysm of abdominal aorta, cholangiocarcinoma	Probable chronic Q fever, no focus discovered

Abbreviations: IgG = immunoglobulin.

^a^ In follow-up sample, the IgG phase I titre was 1:1024.

Table 3 compares the presence of valvulopathy per group of patients. The most common valvular defect was mild mitral regurgitation, mild aortic regurgitation, or mild aortic stenosis.

**Table 3:** Valvulopathy of patients with no serological evidence of a previous *C*. *burnetii* infection, patients with serological evidence of a previous *C*. *burnetii* infection but no chronic Q fever infection, and patients with a chronic Q fever infection.

Heart valve disease		Patients with no serological evidence of a previous *C*. *burnetii* infection	Patients with serological evidence of a previous *C*. *burnetii* infection, no chronic infection	Patients with chronic Q fever
		N = 771	N = 127	N = 6
***Mitral valve total***	n (%)	608 (79)	98 (77)	5 (83)
Mild mitral regurgitation	n (%)	403 (52)	68 (54)	2 (33)
Moderate mitral regurgitation	n (%)	174 (23)	26 (20)	3 (50)
Severe mitral regurgitation	n (%)	20 (3)	3 (2)	0 (0)
Mitral regurgitation with prolapse	n (%)	41 (5)	8 (6)	0 (0)
Mild mitral stenosis	n (%)	14 (2)	1 (<1)	0 (0)
Moderate mitral stenosis	n (%)	2 (<1)	1 (<1)	0 (0)
Severe mitral stenosis	n (%)	1 (<1)	0 (0)	0 (0)
Mitral stenosis with prolapse	n (%)	0 (0)	0 (0)	0 (0)
Mitral paravalvular leakage	n (%)	0 (0)	0 (0)	0 (0)
Mitral prosthetic valve	n (%)	6 (<1)	0 (0)	0 (0)
Mitral valve repair	n (%)	13 (2)	1 (<1)	0 (0)
***Aortic valve total***	n (%)	431 (56)	75 (59)	2 (33)
Mild aortic regurgitation	n (%)	245 (32)	39 (31)	1 (6)
Moderate aortic regurgitation	n (%)	42 (5)	6 (5)	0 (0)
Severe aortic regurgitation	n (%)	0 (0)	1 (<1)	0 (0)
Aortic regurgitation bicuspid valve	n (%)	2 (<1)	0 (0)	1 (17)
Mild aortic stenosis	n (%)	133 (17)	29 (23)	0 (0)
Moderate aortic stenosis	n (%)	63 (8)	8 (6)	1 (17)
Severe aortic stenosis	n (%)	29 (4)	4 (3)	0 (0)
Aortic stenosis bicuspid valve	n (%)	1 (<1)	1 (<1)	1 (17)
Aortic paravalvular leakage	n (%)	1 (<1)	1 (<1)	0 (0)
Aortic prosthetic valve	n (%)	24 (3)	5 (4)	1 (17)

Abbreviations: n = number.

Lastly, we performed univariable logistic regression, in order to determine risk factors for developing chronic Q fever (S3 and S4 Tables). There was only one significant risk factor, namely stenosis of a bicuspid aortic valve. Due to low numbers, we were not able to investigate all possible risk factors that are mentioned in Tables 1 and 3, and were therefore not displayed in S3 and S4 Tables.

## Discussion

We found in this study that 5% of the patients with a valvulopathy who had serological evidence of a previous *C*. *burnetii* infection, had a chronic Q fever infection seven years after the end of the large Q fever epidemic. This percentage is higher than reported after a screening of the general population of a village in the epidemic area, where 34% of the participants had antibodies against *C*. *burnetii*, but only 1% of them had a chronic infection. [15] In contrast, screening studies of patients in areas affected by the Q fever epidemic showed a risk of 8% for those with a history of cardiac valve surgery and 31% for those with an abdominal aortic/iliac aneurysm or aorto-iliac reconstruction. [11, 16] However, these studies were performed closer to the end of the epidemic than our study.

It has been estimated that 703 patients with a known heart valve defect or prosthesis were chronically infected throughout the Netherlands when the epidemic ended in 2010. [17] In the same study, the authors estimated that between 2010 and 2017, 369 of these patients were diagnosed with chronic Q fever or died due to Q fever or another cause. Accordingly, in 2017, an estimated 334 chronic infections were not yet diagnosed in this patient group. Screening for chronic Q fever in patients with valvulopathy, in areas that have experienced moderate to high Q fever incidence, was found to be cost-effective. [17]

In this study, we found that patients with stenosis of a bicuspid aortic valve had a higher risk for chronic infection than patients with other valvulopathies. Having a bicuspid aortic valve was earlier described as a risk factor for chronic Q fever. [18] Interestingly, we did not find that patients with a higher-grade valvulopathy had a higher risk for developing a chronic infection. This corresponds with a study in patients with clinically silent valvulopathies, which showed that patients with a only minor vavulopathy had an increased risk for developing chronic Q fever. [18]

The Dutch National Chronic Q fever database contains clinical data on chronic Q fever patients that are treated in various hospitals. In this database, the infection in most patients has a vascular focus. Of the 323 probable and proven chronic Q fever patients, 153 have a vascular focus, 84 have a cardiac focus, 43 have a combination of cardiac and vascular infection, and 11 have another focus (personal communication Sonja van Roeden, 19-12-2016, [10]). It is noteworthy that in our screening programme of patients with cardiac valvulopathy, two of the six patients detected with chronic Q fever had a vascular focus of infection, rather than endocarditis. However, in France, endocarditis seems to predominate. [7] As earlier described, several possible explanations could account for this difference [19]: differing case definitions for chronic infection, differing virulence of circulating *C*. *burnetii* strains, possible selection bias in a national reference centre, and a lack of specificity in describing valvular defect severity in retrospective reports. [6, 7, 20–23] Lastly, the vascular infections diagnosed in the Netherlands may be an embolic consequence of clinically silent endocarditis, as Million et al suggested. [24] On the other hand, our results reduce the likelihood of one explanation for the discrepancy between the Dutch and French literature. As our systematic screening found more vascular infections than endocarditis, it is unlikely that a lack of systematic screening led to underdiagnoses of endocarditis in the Netherlands.

A major strength of this study was the high participation rate of 95%. We therefore assume that the study population is representative of the general Dutch population with a heart valve defect living in areas that were affected by the Q fever epidemic. The high participation rate may have been influenced by reports on websites and regional media plus a short video on the study that ran continuously in the waiting room area of the Bernhoven hospital.

However, our study also has some limitations. We first screened patients for IgG phase II antibodies against *C*. *burnetii*. Because IgG phase II antibodies wane over the years, some patients with previous acute infection may have been missed, and overall seroprevalence may have been underestimated. [4, 25] Therefore, we might have overestimated the percentage of chronic Q fever patients among those who had serological evidence of a previous *C*. *burnetii* infection. With a lower IgG phase II cut-off value, we would have missed fewer previous infections. However, we chose the cut-off value of IgG phase II of 1:64, to enable comparison of our results with other screening studies, and to minimise false-positive results. [15, 16]. Conversely, we assumed that all patients with chronic Q fever have an IgG phase II titre ≥1:64. [4] Not all valvulopathy patients who attended a cardiologist in the Bernhoven hospital during the study year were invited to participate in this study. The main reason was the high workload of the cardiologists during a reorganisation of the department. A further limitation is that we cannot exclude some form of survival bias in this study, as not all valvulopathy patients who died of an unknown cause during the study year were tested for chronic Q fever. Unfortunately, it is unknown how large this group has been. Therefore, it is possible that we underestimated the chronic Q fever prevalence. Next, patients might have acquired the *C*. *burnetii* infection in the years after the large outbreak. However, the risk was low, as the number of acute Q fever sharply declined after the outbreak. Lastly, we are uncertain whether all six chronic Q fever patients who were diagnosed in the present study were actually infected during the 2007–2010 Q fever epidemic. Acute Q fever remains endemic at very low levels, and chronic Q fever could have been the result of a more recent acute infection.

In conclusion, among 904 patients with a heart valve defect, we diagnosed six cases of chronic Q fever seven years after the end of a large epidemic. Screening of this patient group in other areas affected by the Q fever epidemic may yield additional cases of chronic infection. Diagnosis of chronic Q fever in patients with valvulopathy can be beneficial in preventing future complications of this chronic disease. Results of this study are used as input in a cost-effectiveness study. [26] At this time, a screening program is started in high-risk groups. GPs are now selecting and inviting high risk patients for the screening who live in an area that was affected by the Q fever epidemic.

## Supporting information

S1 Table Dutch consensus guideline on chronic Q fever diagnosis. (DOCX)

S2 Table Characteristics of the study participants, per group of infection status. (DOCX)

S3 Table Univariable risk analysis for chronic Q fever patients versus patients with serological evidence of a previous *C*. *burnetii* infection but no chronic Q fever infection (reference category). (DOCX)

S4 Table Univariable risk analysis for chronic Q fever patients versus patients with no chronic Q fever (patients with serological evidence of a previous C. burnetii infection and patients with serological evidence of a previous C. burnetii infection but no chronic Q fever infection taken together (reference category)). (DOCX)

S1 Dataset Anonymised data of the 904 participants. (XLSX)

S1 File Declaration of the assessment of whether the Medical Research Involving Human Subjects Act (WMO) applies, Medical Ethics Review Committee Brabant. (DOCX)

S2 File Ethical review, Brabant Advisory Committee. (DOCX)
