# Supplementary material for: Risk of chronic Q fever in patients with cardiac valvulopathy, seven years after a large epidemic in the Netherlands
Source: PLoS One. 2019 Aug 22;14(8):e0221247. doi: 10.1371/journal.pone.0221247 (PMC6705838; doi:10.1371/journal.pone.0221247)
Supplement: S1 Table — (DOCX) [file pone.0221247.s001.docx]

**S1 Table.** Dutch consensus guideline on chronic Q fever diagnosis ^a^

| Proven chronic Q fever | Probable chronic Q fever | Possible chronic Q fever |
| --- | --- | --- |
| 1. Positive *C. burnetii* PCR   in blood or tissue ^b^  OR   1. IFA ≥ 1:1024 for   *Coxiella burnetii* phase I IgG AND   - Definite endocarditis according to the modified Duke criteria ^c^   OR   - Proven large vessel or prosthetic infection by imaging studies   (^18^FDG-PET, CT, MRI or AUS) | IFA ≥ 1:1024 for *Coxiella burnetii* phase I IgG  AND one or more of following criteria:   - Valvulopathy not meeting the major criteria of the modified Duke criteria ^c^ - Known aneurysm and/or vascular or cardiac valve prosthesis without signs of infection by means of TEE/TTE, ^18^FDG-PET, CT, MRI or abdominal doppler ultrasound - Suspected osteomyelitis or hepatitis as manifestation of chronic Q fever - Pregnancy - Symptoms and signs of chronic infection, such as fever, weight loss and night sweats, hepato-splenomegaly, persistent raised ESR and CRP - Granulomatous tissue inflammation, proven by histological examination - Immunocompromised state | IFA ≥ 1:1024 for *Coxiella burnetii* phase I IgG without manifestations meeting the criteria for proven or probable chronic Q fever |

Abbreviations: PCR, polymerase chain reaction; IFA, immunofluorescence assay; PET, positron emission tomography; CT, computer tomography; MRI, magnetic resonance imaging; AUS, abdominal ultrasound; TEE, transesophageal echocardiography; TTE, Transthoracic echocardiography, ESR, erythrocyte sedimentation rate; CRP, C-reactive protein.

^a^ Wegdam-Blans MC, Kampschreur LM, Delsing CE, Bleeker-Rovers CP, Sprong T, van Kasteren ME, et al. Chronic Q fever: review of the literature and a proposal of new diagnostic criteria. J Infect, 2012; 64:247-59.

^b^ In absence of acute infection.

^c^ Li JS, Sexton DJ, Mick N, Nettles R, Fowler VG Jr, Ryan T, et al. Proposed modifications to the Duke criteria for the diagnosis of infective endocarditis. Clin Infect Dis, 2000;30:633-8.
