# Supplementary material for: Risk of chronic Q fever in patients with cardiac valvulopathy, seven years after a large epidemic in the Netherlands
Source: PLoS One. 2019 Aug 22;14(8):e0221247. doi: 10.1371/journal.pone.0221247 (PMC6705838; doi:10.1371/journal.pone.0221247)
Supplement: S2 Table — (DOCX) [file pone.0221247.s002.docx]

**S2 Table.** Characteristics of the study participants, per group of infection status ^a^

| **Characteristic** | | **Patients with no serological evidence of a previous *C. burnetii* infection** | **Patients with serological evidence of a previous *C. burnetii* infection, no chronic infection** | **Patients with chronic Q fever** |
| --- | --- | --- | --- | --- |
| Gender | n/N (% male) | 397/771 (51) | 85/127 (67) | 5/6 (83) |
| Age | Median (min – max) (years) | 73 (26-95) | 72 (39-89) | 78 (61-83) |
| *Comorbidity* | | | | |
| Diabetes | n/N (%) | 138/755 (18) | 19/125 (15) | 0/6 (0) |
| COPD | n/N (%) | 66/755 (9) | 13/125 (10) | 1/6 (17) |
| Asthma | n/N (%) | 45/755 (6) | 4/125 (3) | 0/6 (0) |
| Impaired kidney function or chronic kidney disease ^b^ | n/N (%) | 207/755 (27) | 30/125 (24) | 1/6 (17) |
| Stroke | n/N (%) | 52/755 (7) | 8/125 (6) | 0/6 (0) |
| Hematologic cancer ^c^ | n/N (%) | 3/755 (<1)) | 1/125 (1) | 0/6 (0) |
| Cancer, other than hematologic cancer ^c^ | n/N (%) | 79/755 (10) | 11/125 (9) | 0/6 (0) |
| Autoimmune disease | n/N (%) | 97/755 (13) | 10/125 (8) | 0/6 (0) |
| HIV | n/N (%) | 0/755 (0) | 0/125 (0) | 0/6 (0) |
| Vascular prosthesis of the large body vessels ^d^ | n/N (%) | 25/755 (3) | 4/125 (3) | 1/6 (17) |
| Vascular abnormality of the large body vessels ^e^ | n/N (%) | 73/755 (10) | 11/125 (9) | 2/6 (33) |

Abbreviations: n=Number, N=total number

^a^ 18 Participants gave no permission to collect data from the electronic patient record.

^b^ eGFR (MDRD) in majority of tests in recent years smaller than 60.

^c^ Cancer present in last five years before inclusion.

^d^ Vascular prosthesis of the aorta, femoral artery, or common iliac artery.

^e^ Aneurysm or vascular dilatation of the aortic arch (>29mm) or, ascending aorta (>40 mm) described in echocardiographic report or dilatation of abdominal aorta, femoral artery, or common iliac artery mentioned in the electronic patient record.
