# Supplementary material for: Risk of chronic Q fever in patients with cardiac valvulopathy, seven years after a large epidemic in the Netherlands
Source: PLoS One. 2019 Aug 22;14(8):e0221247. doi: 10.1371/journal.pone.0221247 (PMC6705838; doi:10.1371/journal.pone.0221247)
Supplement: S3 Table — (DOCX) [file pone.0221247.s003.docx]

**S3 Table.** Univariable risk analysis for chronic Q fever patients versus patients with serological evidence of a previous *C. burnetii* infection but no chronic Q fever infection (reference category).

| **Characteristic** | **Odds ratio (95% CI)** | **P-value** |
| --- | --- | --- |
| Mild mitral regurgitation (yes vs. no) | 0.43 (0.08 – 2.45) | 0.34 |
| Moderate mitral regurgitation (yes vs. no) | 3.89 (0.74 – 20.38) | 0.11 |
| Mild aortic regurgitation (yes vs. no) | 0.45 (0.05 – 3.99) | 0.47 |
| Moderate aortic stenosis (yes vs. no) | 2.98 (0.31 – 28.60) | 0.35 |
| Stenosis of bicuspid aortic valve (yes vs. no) | 25.20 (1.37 – 463.64) | 0.03 |
| Aortic prosthetic valve (yes vs. no) | 4.88 (0.48 – 49.95) | 0.18 |
| Age (≥75 years vs. <75 years) | 2.54 (0.45 – 14.35) | 0.29 |
| Gender (male vs. female) | 2.47 (0.28 – 21.82) | 0.42 |
| COPD (yes vs. no) | 1.72 (0.19 – 15.91) | 0.63 |
| Impaired kidney function (yes vs. no) | 0.63 (0.07 – 5.64) | 0.68 |
| Vascular prosthesis of the large body vessels (yes vs. no) | 6.05 (0.57 – 64.51) | 0.14 |
| Aneurysm large body vessels (yes vs. no) | 5.18 (0.85 – 31.56) | 0.07 |

Abbreviations: CI = confidence interval.

Due to low numbers, we were not able to investigate all possible risk factors that are mentioned in Table 1 and 3. Here we show the results of the characteristics for which we could perform a risk factor analysis.
