# Supplementary material for: Risk of chronic Q fever in patients with cardiac valvulopathy, seven years after a large epidemic in the Netherlands
Source: PLoS One. 2019 Aug 22;14(8):e0221247. doi: 10.1371/journal.pone.0221247 (PMC6705838; doi:10.1371/journal.pone.0221247)
Supplement: S4 Table — (DOCX) [file pone.0221247.s004.docx]

**S4 Table.** Univariable risk analysis for chronic Q fever patients versus patients with no chronic Q fever (patients with serological evidence of a previous *C. burnetii* infection and patients with serological evidence of a previous *C. burnetii* infection but no chronic Q fever infection taken together (reference category)).

| **Characteristic** | **Odds ratio (95% CI)** | **P-value** |
| --- | --- | --- |
| Mild mitral regurgitation (yes vs. no) | 0.45 (0.08 – 2.49) | 0.36 |
| Moderate mitral regurgitation (yes vs. no) | 3.49 (0.70 – 17.43) | 0.13 |
| Mild aortic regurgitation (yes vs. no) | 0.43 (0.05 – 3.72) | 0.45 |
| Moderate aortic stenosis (yes vs. no) | 2.33 (0.27 – 20.21) | 0.44 |
| Aortic prosthetic valve (yes vs. no) | 5.99 (0.68 – 52.95) | 0.11 |
| Age (≥75 years vs. <75 years) | 2.56 (0.47 – 14.03) | 0.28 |
| Gender (male vs. female) | 4.32 (0.50 – 37.09) | 0.18 |
| COPD (yes vs. no) | 2.03 (0.23 – 17.6) | 0.52 |
| Impaired kidney function (yes vs. no) | 0.54 (0.06 – 4.67) | 0.58 |
| Vascular prosthesis of the large body vessels (yes vs. no) | 5.87 (0.66 – 51.85) | 0.11 |
| Aneurysm large body vessels (yes vs. no) | 4.74 (0.86 – 26.26) | 0.08 |

Abbreviations: CI = confidence interval.

Due to low numbers, we were not able to investigate all possible risk factors that are mentioned in Table 1 and 3. Here we show the results of the characteristics for which we could perform a risk factor analysis.
