# Supplementary material for: Risk of chronic Q fever in patients with cardiac valvulopathy, seven years after a large epidemic in the Netherlands
Source: PLoS One. 2019 Aug 22;14(8):e0221247. doi: 10.1371/journal.pone.0221247 (PMC6705838; doi:10.1371/journal.pone.0221247)
Supplement: S1 File — (DOCX) [file pone.0221247.s006.docx]

National Institute for Public Health and the Environment (RIVM)

Ms. M. de Lange

PO Box 1

3720 BA Bilthoven (NL)

| Phone | +31 (0)13-5398006/8027 |
| --- | --- |
| E-mail | [info@metcbrabant.nl](mailto:info@metcbrabant.nl) |
| Our reference | METC Brabant/16.023 |
| Date | 01/13/2016 |

**Declaration of the assessment of whether the Medical Research Involving Human Subjects Act (WMO) applies.**

|  |  | **METC #** | **P1551** |
| --- | --- | --- | --- |
| **Title of Study**: | **Q-Cor**  Better recognition of chronic Q fever in patients with heart valve problems.  **P1551** | | |

Dear Ms. De Lange,

In the meetings of November 2, 2015, December 14, 2015, and January 11, 2016, the Brabant MERC used the information provided by you for its discussion of whether the execution of the proposed above-mentioned study does or does not fall within the scope and associated obligations of the Medical Research Involving Human Subjects Act.

In that assessment, the Brabant MERC had the following information that had been provided by you:

- A1 Letters from Ms. M. de Lange dated 10/01/2015 and 11/24/2015
- A1 Letter with further questions to the Brabant MERC dated 11/03/2015
- A1 Response letter dated 11/23/15 from Ms. M. de Lange
- C1 Study Protocol, version 2, dated 11/23/2015 + tracked changes;
- E1 Patient Letter version 2, dated 11/23/2015 + tracked changes;
- E2 Informed Consent Form, version 2, dated 11/23/2015 + tracked changes;
- G2 Proof of liability insurance coverage, dated 01/13/2015;
- H1 Résumé of the independent expert, undated;
- I2 Study Declaration, dated 09/28/2015;
- I3 Résumé of the principal investigator, undated;
- K1 Copy of assessment by other organizations, dated 06/10/2015;

**Based on the information you provided, the Brabant MERC determined that your proposed study does not fall within the scope of the Medical Research Involving Human Subjects Act.**

**Explanation:**

This is an offer to study people in a high-risk area with an elevated risk of chronic Q fever in order to be able to offer them appropriate treatment, where there is no scientific objective, research question, and study setup because a validated research method is being used and a regular range of treatments is available.

The Brabant MERC explicitly points out that the assessment carried out and the declaration issued on the subject only concern the question of whether or not your proposed study (or the study to be carried out under your responsibility) falls under the scope of the Medical Research Involving Human Subjects Act.

The assessment and this declaration do not constitute a substantive assessment by the Brabant MERC of the relevance, quality, and conformity with applicable legislation, regulations and ethical requirements for this study.

**Please refer to the enclosed advice from the Brabant Advisory Committee for this.**

Kind regards,

On behalf of the Brabant MERC

[signature]

H.W.M. van Heertum

Official Secretary
