# Supplementary material for: Risk of chronic Q fever in patients with cardiac valvulopathy, seven years after a large epidemic in the Netherlands
Source: PLoS One. 2019 Aug 22;14(8):e0221247. doi: 10.1371/journal.pone.0221247 (PMC6705838; doi:10.1371/journal.pone.0221247)
Supplement: S2 File — (DOCX) [file pone.0221247.s007.docx]

Ms. M. de Lange, RIVM

PO Box 1

3720 BA Bilthoven (NL)

| Phone | +31 (0)13-5398006/8027 |
| --- | --- |
| E-mail | [info@metcbrabant.nl](mailto:info@metcbrabant.nl) |
| Our reference | AC Brabant/16.022 |
| Date | 01/13/2016 |

ADVICE

|  |  | **METC #** | **P1551** |
| --- | --- | --- | --- |
| **Title of Study** | **Q-Cor**  Better recognition of chronic Q fever in patients with heart valve problems.  **P1551** | | |

Dear Ms. De Lange,

In its meeting of January 11, 2016, the Brabant Advisory Committee discussed the following documents based on the information you provided:

For that assessment, the Brabant Advisory Committee had the following information that had been provided by you:

- A1 Letters from Ms. M. de Lange dated 10/01/2015 and 11/24/2015
- A1 Letter with further questions to the Medical Ethics Committee of Brabant (*METC Brabant*) dated 11/03/2015
- A1 Response letter dated 11/23/15 from Ms. M. de Lange
- B1 General Assessment and Registration form (ABR form), version 4.0, dated 11/23/2015;
- C1 Study Protocol, version 2, dated 11/23/2015 + tracked changes;
- E1 Patient Letter version 2, dated 11/23/2015 + tracked changes;
- E2 Informed Consent Form, version 2, dated 11/23/2015 + tracked changes;
- G2 Proof of liability insurance coverage, dated 01/13/2015;
- H1 Résumé of the independent expert, undated;
- I2 Study Declaration, dated 09/28/2015;
- I3 Résumé of the principal investigator, undated;
- K1 Copy of assessment by other organizations, dated 06/10/2015;

**Based on that information, the Brabant Advisory Committee came to the conclusion that the study meets the rules where applicable as laid down in the Dutch Data Protection Act and Medical Treatment Contracts Act, the Code of Conduct for Health Research, and the Code of Conduct for Responsible Use.**

**Moreover, there are no medical ethical objections against this study being carried out.**

The Committee would like to wish you every success in carrying out the study.

On behalf of the Brabant Advisory Committee,

[signature]

H.W.M. van Heertum

Official Secretary
